# Wonderful Life: An Interview with Herb Boyer

**DOI:** 10.1371/journal.pgen.1000653

**Published:** 2009-09-25

**Authors:** Jane Gitschier

**Affiliations:** Departments of Medicine and Pediatrics, Institute for Human Genetics, University of California San Francisco, San Francisco, California, United States of America

Once upon a time, not so very long ago, before restriction enzymes were ordered from a New England Biolabs catalog and vectors arrived in neat packages from Promega, and before molecular biologists added patents or a company to their CV, there was Herb Boyer. One can almost define the revolution in molecular genetics by Herb's story alone—the discovery of the iconic restriction enzyme EcoR1 and the definition of its sticky ends, the collaboration with Stan Cohen that produced recombinant DNA, and the genesis of the enduring gold standard in biotechnology, Genentech.

I had been fascinated by Herb's story for many years, as I myself had the good fortune to do a post-doc at Genentech in the early 1980s. Much has been written about Herb Boyer (Image 1), so I chose not to talk with him about his role in the founding of Genentech in 1976, nor the landmark Boyer-Cohen patent, nor the mid-1970s moratorium on recombinant DNA research. Instead, I was interested in what came before all of that—how Herb developed as a scientist, how he become interested in restriction enzymes and in vitro recombination—and by what came later. I'm sure you'll agree, this is equally rich reading.

**Figure:**
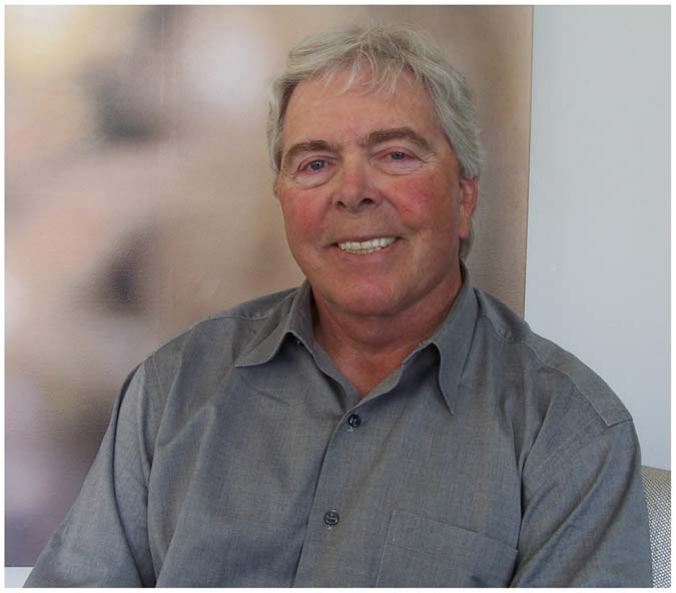
Herb Boyer.

**Gitschier:** I know you grew up in the little town of Derry, Pennsylvania. What role do you think your upbringing played in some of the choices you made in your life?

**Boyer:** My mother graduated from high school, and she immediately married my father at the age of 18. My dad was 12 years older than she. My dad left school after the 8th grade and went to work. He came from a large family with six siblings. He was the oldest boy, so the story was he quit school to help support his family.

I'm trying to write my memoirs now, so I've been thinking a lot about my early childhood. My upbringing was in a town of about 3,000 people and the principal industries were the railroad and a Westinghouse manufacturing facility. I went to a small school, only 32 in my graduating class, and I loved sports and the outdoors. I used to hunt and fish with my father.

**Gitschier:** Where did your father work?

**Boyer:** He worked for the Pennsylvania Railroad. My father never owned a car, never had a driver's license. He'd walk to work at the dispatch station, which was about a hundred yards away from our house. He was a brakeman on the freight trains and would live on a caboose for a couple of days at a time. I always thought that was rather romantic. He worked in terrible winter weather and stifling summer heat. By the time he retired, at age 72, he was a conductor, and at that point he was making $12,500 a year. That was my starting salary at UCSF [University of California San Francisco].

High school for me was football, basketball, baseball, girls, hunting, and fishing. And I worked at odd jobs. I dug ditches, mowed lawns for 50 cents, painted houses, distributed door-to-door advertisements, and of course I was a newspaper boy.

**Gitschier:** Was your father encouraging about your staying in school and going to college?

**Boyer:** I can never remember getting encouragement from my mother or father to go beyond a high school education, though they weren't opposed to it. I just knew I had to get out of Derry, and that was the only way I knew how to do it!

**Gitschier:** Your father lived at least 10 years after you became an assistant professor at UCSF.

**Boyer:** Yes, he was 83 or 84 when he died.

**Gitschier:** So, he watched this incredible progression in your life. What was that like for you and for him?

**Boyer:** I'm not sure he had much of an appreciation for what I did.

**Gitschier:** Did he ask you about it?

**Boyer:** Not a lot. He was a very quiet man. My dad was just happy I didn't turn out to be a ne'er-do-well. I had a job and I had kept it for a few years and that was good enough for him.

But Jane, how could my parents relate to this? They didn't know what science was or anything about experimental procedures. This is true of many people today, even with substantial educational backgrounds.

**Gitschier:** Let's shift gears. I'd like to talk to you about restriction and modification.

**Boyer:** Four or five years ago, Stan [Cohen] and I received the Sir Run Run Shaw Prize in Hong Kong. In my acceptance speech, I recounted some history of restriction and modification and all the little threads that appear to have twisted my career—binary events that seem improbable to have happened. If this event didn't happen, what would have happened? But it happened, so you go to another event. What is that movie with Jimmy Stewart where he's about to throw himself off the bridge?

**Gitschier:** “It's a Wonderful Life.”

**Boyer:** It's sort of like that—I can give you a few examples. I went to a Benedictine college and I took an elective physiology course taught by Father Joel. We had a brand new, shiny textbook with a blue and white cover. Each of us was assigned a chapter, and we had to give a seminar on it. Which one did I get? “The Structure of DNA.” This was 1957, and the buzz of DNA was just getting into the textbooks. And I had this fascination with genetics—classical genetics—*Drosophila*, corn, and bacteriology. I was really taken with the Watson-Crick structure of DNA and this started my fascination with the heuristic value of the structure.

Then I had to decide what to do. I went to the University of Pittsburgh Medical School for an interview with this tough old biochemist. And he said, “Well, if you get into medical school, how are you going to pay for it?” And I looked at him and I said, “You mean I have to pay for it?”

But I didn't get in, which was hard on my ego.

**Gitschier:** So there is binary point number one.

**Boyer:** You see! My grades were not that terrific, but remember that was before grade inflation. I got a D in metaphysics, and that didn't help! I was taking a liberal arts program. I got A's in math, logic, etc., but all that other stuff—Chaucer—ouhhh. Someone suggested going to grad school for a couple of years, improving my grades, and reapplying to medical school.

So, I arrived at the University of Pittsburgh at the same time as a professor studying bacterial genetics and gene regulation—Ellis Engelsberg. This is 1958. You know, in 1958 the genetic code wasn't known, and nobody knew anything about messenger RNA, and protein synthesis was still speculative. If you think about what has happened in 50 years—it's unbelievable.

Anyhow, I get singled out because I'm a new graduate student with an interest in bacterial genetics. So I get a chance to work in Engelsberg's lab on the genetic control of the L-arabinose metabolic pathway in *E. coli*. Ellis had recruited Roger Weinberg as an assistant professor and collaborator. Roger had found an L-arabinose mutant defective for the epimerase enzyme. In the presence of L-arabinose, the cell accumulates phosphorylated ribulose, which inhibits growth of the cell. So it's an easy way to select for mutants in all the preceding genes of the pathway.

So Roger Weinberg decides that this should be my project! I had to select mutants induced by mutagens that theoretically could induce specific base pair changes in the DNA. This could be challenged by reversion of the mutation with another mutagen to wild type. After mapping the mutations and then doing amino acid substitution analyses, we would solve the genetic code, the Holy Grail of genetics!!!

That was the plan! What the hell was I thinking? Why didn't I challenge these guys?

The project required that I map arabinose mutants by the most inefficient way to do recombination that you can ever imagine—P1 phage transduction. At a low frequency, the phage can incorporate small fragments of DNA that when injected into a cell can lead to recombination with the cellular chromosome. Anyway, if you get yields of 10^9^ phage per ml you were doing well, and the frequency of recombination is maybe one in a thousand.

So, I started mapping a limited region of an arabinose gene, doing forward and reverse mutational analyses, and I was getting worried, “Am I ever going to get out of here?” But, even before I got too far along on this project, the genetic code is cracked by biochemical means! What a blow! But I continued on with the project for some reason I can't remember.

I did decide on my own, though, that P1 transduction was not the way to do fine structure mapping. In those days it didn't take too much to know the literature, and I knew the literature cold. I was familiar with all the latest work on Hfrs and sexuality in bacteria, so I felt this system [bacterial conjugation] would provide higher recombination frequencies. So I wrote to Ed Adelberg at Yale and asked him if he would send me a couple of Hfr strains. And Ed, being such a super guy, sent them right away.

I started doing the crosses. The strain I had been using was *E. coli* B/r. The Hfr strains, of course, were K12s, so I had to start by asking whether K12 would actually mate with the B/r—no one had ever done it before. I started out by crossing Hfr K12 to the B/r strain and comparing that with K12 to K12, as a standard. I found there was a substantial reduction in frequency of recombination [in the K12 to B/r strain], and the linkage of the various genes was also reduced.

So I started to do some backcrosses and I found out that some progeny of the cross did not exhibit these anomalous genetic results. I mapped the alleles to a region near the arabinose operon. Coincidence!

**Gitschier:** Did you publish that result?

**Boyer:** No, not at that time. I managed to write an acceptable Ph.D. dissertation with the other data. However, by that time I was very interested in trying to explain my observation and became more intrigued with plasmids, conjugation, and bacterial sexuality. Ed Adelberg had written a book and papers and reviews on these subjects. So I wrote to Ed and applied for a postdoctoral fellowship with him, and he said, “Yeah, come on!”

**Gitschier:** Did Ed know about your results about K12 and B/r?

**Boyer:** Not at the time, but upon arrival in his lab I described my results and he encouraged me to continue my experiments as well as a couple of other projects he suggested. But my heart was in trying to explain my observations.

Ed had a fairly small and close-knit group and we would work in the evenings and chat. Not long before I arrived, Werner Arber and Daisy Dussoix demonstrated that the restriction and modification of DNA, a relatively ignored bacterial phenomonology used for typing clinical bacterial strains, was associated with methylation of DNA and a site-specific endonucleolytic activity. One evening, a graduate student in the lab, Noel Bouck, and I were discussing the paper and she said, “I really think that the anomalies you're seeing are due to the same thing.”

So I took out the stains in which I had changed the specificities, and did the lambda phage restriction analyses. And boom, boom, boom, it just lined up. I had mapped the restriction and modification alleles of *E. coli* K12 and B.

I became interested in pursuing it further because of the predicted enzymatic specificity. There were maybe three laboratories in the world working on restriction and modification at that time. I wanted to purify and characterize these enzymes because I thought it would be a great way to study site-specific interactions between proteins and DNA. Unlike repressors, restriction and modification enzymes involved two proteins [the endonuclease and the methylase] that have to recognize the same sequence, and I thought that it would be pretty cool to have two different ways of looking at it.

So, the last year at Yale, I experimented with some way to assay for the K12 and B restriction enzymes. And then I headed to California [UCSF] where we finally settled on an assay based on the sedimentation coefficient of radioactive lambda DNA [sigh!]. This is pre-gels—what a mess! We had a 6-hour run, and we'd do three runs a day. You'd take the little centrifuge tubes, punch them at the bottom, collect the contents drop-by-drop on little squares of filter paper hung on a pin on a piece of styrofoam, dry the papers, and put them into the scintillation counter. It was SO bad. And we couldn't find any activity at all!

Then Matt Meselsohn and Bob Yuan at Harvard—I can't remember the rationale—they threw S-adenosyl methionine and ATP into the reaction and got activity! So we did that, too, and we started purifying those enzymes.

**Gitschier:** What year roughly are we?

**Boyer:** The period of 1966–1969. We went on to purify the B restriction endonuclease and began experiments to determine the sequence at the cleavage site, which we assumed would be the recognition site. So we kept labeling the 5′ end of cleaved DNA molecules and we always ended up with equal mixtures of four nucleotides. We never even thought that there would be an endonuclease that would bind at a specific site and then move! I was so disappointed.

**Gitschier:** But at some point, you make a switch, and you start working on a different restriction enzyme.

**Boyer:** Well, here comes one of the most bizarre little binary points in life! We found out that these [Type I] endonucleases aren't cutting at a unique site. We used a small phage DNA intermediate and cleaved it with the B endonuclease. By sedimentation analysis it looked like it had a molecular weight of a linear fragment, and that was what threw us off.

**Gitschier:** Why?

**Boyer:** Well, we thought it had one site, and you assume it is always cleaving at the same site! Apparently it was cleaving, on average, one site, but not the recognition site. Stu Linn at Berkeley demonstrated, by electron microscopy, that what appeared to be linear products of the B endonuclease could be [denatured and] reannealed as circular molecules.

So I thought, this is it! This is the end. We're not going to be able to determine the [recognition] site with the technology available at the time.

But there was literature, mainly from Japan, demonstrating that bacteria carrying drug resistance factors often had genes for the restriction and modification of DNA with different specificities. So we decided to investigate these enzymes. I had a graduate student who had a degree in clinical microbiology and had experience working in a hospital medical microbiology lab, a wonderful guy named Bob Yoshimori.

I asked Bob to go to the clinical lab at the UCSF hospital and get a slew of multiple drug-resistant *E. coli*. He came back with 36 or so *E. coli* isolates that had multiple drug resistance. And of those, we found eight or ten with restriction and modification activity as determined by phage specificity analyses. We transferred the plasmids into our K12 strains [restriction mutants]. Most of the specificities were like the one that had been reported previously, namely RII [named after the RII plasmid], but we found one that was unique, and that was EcoRI.

We found out later that the EcoRI plasmid came from a woman who was admitted to the hospital with an *E. coli* urinary tract infection that was resistant to multiple antibiotics. Now, the RI endonuclease was never found anywhere else for 15 years, and then it was found in a freshwater microorganism. It just had the same specificity as RI.

**Gitschier:** Wow, you should write her a thank you note.

**Boyer:** Well, I wish I had her name. But how would I explain this to her? She's probably not even alive today.

**Gitschier:** And it could backfire. She could sue!

**Boyer:** Well, I've thought of that too!

**Gitschier:** OK, you've got EcoRII and EcoRI.

**Boyer:** We purified the restriction and modification enzymes of both specificities. We were so thrilled with the first centrifugation experiments. We digested lambda DNA and we had these clear-cut separations of fragments in the sedimentation analysis. And the patterns were different from each other. We went on to determine the sequence of the cleaved and methylated sites of the RI and RII enzymes. This gave us a belated sense of achievement given our prior experiments.

**Gitschier:** Was it the *Haemophilus influenza* work that was going on around the same time that made you think there might be some enzymes out there for which you could find a cleavage site?

**Boyer:** We knew about the work of Ham Smith and Dan Nathans. And that was SO frustrating because there they got that sequence and we had been working for at least a year! By that time Howard Goodman had come to UCSF. He had experience sequencing RNA molecules, and so we naturally started a collaboration on the sequence of the cleaved and methylated sites. At that point, we knew that RI didn't cleave as frequently as all of the other enzymes.

And then Paul Berg called. He had heard about this enzyme and wanted to know if he could get some. I said, “Sure.” Bob gave someone from his lab enough enzyme to last a lifetime, and Berg gave it to a couple of his post-docs and graduate students. And it was actually Janet Mertz and Ron Davis, an assistant professor at Stanford, who cleaved SV40 with EcoRI and then looked at it under an electron microscope. They found that the cleaved DNA would circularize at low temperatures, and that's the first evidence for the enzyme generating cohesive ends.

Paul told us this while we were working on the sequence of the cleaved end. We already knew the 5′ nucleotide, so I went to Mike Bishop and said, “Mike, we need some reverse transcriptase,” and we filled in the single-stranded part of the end and got the sequence overnight. It was another eureka moment! There was a young medical student [Judy Aldrich] working on a summer research project and she and I looked at the results the next morning, a Saturday—it's GAATTC!

**Gitschier:** So the RI sticky ends led directly to in vitro recombination. Let's talk about how that took off.

**Boyer:** My own interest in recombination goes back to graduate school. It was almost an article of faith that DNA would break and exchange strands at any point along the polynucleotide chain. I remember reading Dale Kaiser's papers on the cohesive ends of lambda, and musing about restriction enzymes and Sanger's techniques for determining the sequences of proteins with two-dimensional chromatography. I was thinking if you could break down DNA with these different enzymes, given their specificities, you might be able to separate smaller fragments and sequence them somehow.

I got an invitation to go to Hawaii around 1971 for an East-West conference on plasmids and drug-resistant factors. Stanley Falkow was there—a great guy, known him since my days at Yale. While talking to Stan about our enzyme work, I said, “Stan—you know those plasmids you work with—we can take these things apart and separate the fragments and maybe look at where these resistance genes are.” And he says, “You go talk to Stan Cohen—he's interested in that.”

So Stan Cohen and I get together and learn of our mutual interests in plasmids and in vitro recombination. He had just described pSC101, which conferred resistance to tetracycline, and we realized it might be of significant value given its small size. And just as importantly, he had become aware of a scientist at the University of Hawaii who could transform *E. coli* with DNA. It made all the difference in the world to our thinking. So we agree that we would see if we could cleave the pSC101 molecule with EcoRI and use it for a collaborative recombination experiment.

Then another stroke of good luck. I was scheduled to go to Cold Spring Harbor to give a talk. I get picked up at the airport by Joe Sambrook and Phil Sharp, and they immediately take me into a darkroom adjacent to their laboratory and show me an agarose gel that had been run with cleaved adenovirus DNA and stained with ethidium bromide. It was one of the most exciting things I could have looked at, and I said, “Thank you, lord!” Because prior to that, we'd have to analyze cleaved DNA fragments by polyacrylamide gel analysis. We would put the gel in a small metal tube and then mechanically push it into a guillotine-like device and slice small fragments into scintillation vials. It would go chop, chop, chop, and invariably pieces would fly across the room and we'd be down on the floor looking for slices. It was like looking for a fallen contact lens. It was so laborious and there was such variation in the tritium counts. So I knew immediately that all the laborious work we had done, we don't have to do anymore!

We immediately found, using the Sambrook/Sharp technique, that pSC101 was cleaved once with EcoRI. Stan sent the DNA up, and we cleaved it and did the ligation.

**Gitschier:** What was the other entity?

**Boyer:** Stan had another plasmid with two different antibiotic resistance genes. Annie Chang, Stan's technician who lived in San Francisco, transported the DNA back and forth between our labs. Stan's lab would send us the plasmid DNA, we would do the enzymatic treatments, Stan's lab would do the transformation and selection, Annie would bring back the plasmids, and we would analyze them by cleavage and gel analysis of the fragments.

That was another eureka moment. Bob Helling, a fellow graduate student of mine from University of Pittsburgh who was doing a sabbatical in my lab, and I went to look at the gels in the darkroom, and there it was. It actually brought tears to my eyes, it was so exciting, and I knew what we had done had a lot of potential.

**Gitschier:** What kind of potential?

**Boyer:** A lot of my mentors and colleagues were leaving microbial systems to study higher order cells, because “everything there was to know about bacteria was known.” But they were frustrated, because they had no hope to isolate single genes or fragments of genes from the chromosomes of “higher” organisms. So when I looked at those gels, I knew we'd be able to isolate any piece of DNA that was cut with EcoRI, regardless of where it came from.

**Gitschier:** I was just re-reading “Invisible Frontiers” [about the race to clone the human insulin gene, by Stephen S. Hall].

**Boyer:** Great book.

**Gitschier:** Yes, and what I didn't appreciate was that October 14, 1980, was the day Gilbert, Sanger, and Berg won the Nobel prize AND the day Genentech went public. That must have been a very interesting day for you.

**Boyer:** Yeah. We were gathered at the company to follow the reaction to our IPO [initial public offering] and someone came into the room with the morning [San Francisco] *Chronicle*. And the headline was “Genentech Jolts Wall Street” and underneath is a photo of Paul Berg, “Berg Wins Nobel Prize”.

**Gitschier:** Many people have speculated about why it is you and Stan Cohen have never won a Nobel Prize, but I don't know that you've ever talked about that publicly. Are you going to address this in your memoir?

**Boyer:** I will, and I don't mind talking about it with you.

It is not for me to decide whether I should or should not win a Nobel Prize. I've received many prizes and honors and I am indeed grateful for the recognition. You can imagine from what I've said about my boyhood, that I never would have expected to do what I've done. I wanted to do something important—I didn't know what it would be, but I planned to work hard and see what I could do. I had no foresight that this would be what it was.

**Gitschier:** When you say “that this would be what it was”, are you referring to the scientific work, or are you referring to Genentech?

**Boyer:** Both, I don't separate them. I don't know how I could separate those two events in my life.

Disappointed at times? Yeah. But I've been through quite a few periods in my life where I've had strong emotional reactions to one thing or another. All of the criticisms and rebuffs from colleagues that came when I started Genentech, and prior to that attacks on recombinant DNA technology. One of the most difficult periods for me was when my UCSF colleagues were fairly critical; I was the subject of an Academic Senate investigation. Gee, I thought what I was doing was a pretty good thing, and you'd think I was a criminal! That I found to be much more difficult than not getting a Nobel Prize. All in all, these experiences can be of great value to your outlook on life.

I have been rewarded, and I am so lucky, Jane. And I'm so grateful.

